# Organelle-Targeted Fluorescent Probes for Sulfane Sulfur Species

**DOI:** 10.3390/antiox12030590

**Published:** 2023-02-27

**Authors:** Biswajit Roy, Meg Shieh, Geat Ramush, Ming Xian

**Affiliations:** Department of Chemistry, Brown University, Providence, RI 02912, USA

**Keywords:** sulfane sulfur, fluorescent probe, organelle, chemistry

## Abstract

Sulfane sulfurs, which include hydropersulfides (RSSH), hydrogen polysulfides (H_2_S_n_, n > 1), and polysulfides (RS_n_R, n > 2), play important roles in cellular redox biology and are closely linked to hydrogen sulfide (H_2_S) signaling. While most studies on sulfane sulfur detection have focused on sulfane sulfurs in the whole cell, increasing the recognition of the effects of reactive sulfur species on the functions of various subcellular organelles has emerged. This has driven a need for organelle-targeted detection methods. However, the detection of sulfane sulfurs, particularly of RSSH and H_2_S_n_, in biological systems is still a challenge due to their low endogenous concentrations and instabilities. In this review, we summarize the development and design of organelle-targeted fluorescent sulfane sulfur probes, examine their organelle-targeting strategies and choices of fluorophores (e.g., ratiometric, near-infrared, etc.), and discuss their mechanisms and ability to detect endogenous and exogenous sulfane sulfur species. We also present the advantages and limitations of the probes and propose directions for future work on this topic.

## 1. Introduction

Biological sulfane sulfurs (S^0^), including hydropersulfides (RSSH), polysulfides (RSS_n_SR), hydrogen polysulfides (H_2_S_n_, n ≥ 2), and protein-bound elemental sulfurs (S_8_), have become increasingly recognized as important reactive sulfur species (RSS) with distinct functions in redox biology that are closely linked to hydrogen sulfide (H_2_S) signaling [1]. Sulfane sulfurs are sulfur atoms with six valence electrons and no charge that are covalently bonded to other sulfur atoms. Significantly, sulfane sulfurs have been discovered to influence various physiological and pathological processes, including activating the transient receptor potential ankyrin 1 (TRPA1) channel, relaxing vascular smooth muscles, mediating neurotransmission, and regulating inflammation [2,3,4,5]. Yet, due to their instabilities, sulfane sulfurs such as RSSH and H_2_S_n_ are understudied despite their active involvement in redox signaling. Considering the importance of sulfane sulfurs in biological systems, the development of detection methods for these species is important to better understand their biological mechanisms of action and potential therapeutic applications. 

Some of the most popular detection methods for sulfane sulfurs or other biologically important analytes are fluorescence spectroscopy and fluorescence microscopy [6,7,8,9,10,11,12,13,14,15]. These methods involve the usage of fluorescent probes, which are important tools in the study of biological systems because they allow researchers to visualize and track specific molecules or processes within cells and tissues. By emitting light when excited by a specific wavelength of light, fluorescent probes allow scientists to detect and even quantify the presence of specific molecules in real time [13,14,15]. Thus, fluorescent probes can answer fundamental questions regarding the production and mechanisms of action for sulfane sulfurs in biological samples, making these probes essential for medical diagnosis, treatment, and basic biomedical research. 

Most reported fluorescent probes and studies for sulfane sulfur detection examine cellular sulfane sulfur levels rather than those in subcellular microenvironments. Yet, organelles are specialized subunits within cells that perform specific functions that are essential for the overall health and survival of the cell. In events of stress or malfunction, disease can result. For example, the mitochondria are involved in many critical processes, including the regulation of cell signaling and differentiation, cell death pathways, and the cell cycle [16]. Mitochondrial oxidative damage has been found to contribute to a wide range of human disorders, including ischemia-reperfusion injury and aging-associated dysfunction [17]. While studies have found that H_2_S offers cardioprotective effects by preserving mitochondrial function, sulfane sulfurs are less well-studied. It has been reported, however, that the majority of bound sulfane sulfurs in cells are in the mitochondria, suggesting the importance of this organelle in maintaining cardiovascular homeostasis [18]. It is also known that the mitochondrial enzyme sulfide quinone oxidoreductase (SQOR) rapidly converts H_2_S into sulfane sulfurs (persulfides and polysulfides) which are then stored in the mitochondria until they are released in response to physiological signals [19]. Considering that this organelle has been found to play key roles in diseases and sulfane sulfurs have been found as actual signaling species in a range of biological activities previously attributed to H_2_S, an accurate, sensitive, and real-time method for detecting sulfane sulfurs in the mitochondria is essential to understand their mechanisms. 

Other subcellular organelles also have specific functions that contribute to the operation of a cell and can result in disease in the event of dysfunction. For example, lysosomes are single-layered membrane organelles that are responsible for cellular waste digestion and contain acidic environments and hydrolases. RSS plays a role in the regulation of lysosomal activity and membrane permeability, thus affecting many biological processes [20]. The rough endoplasmic reticulum (ER) is responsible for protein synthesis, while the smooth ER is primarily involved in calcium signaling, lipid synthesis, and carbohydrate metabolism. ER stress and protein misfolding have been associated with diseases, including myocardial ischemia-reperfusion (MI/R) injury, cardiomyopathy, heart failure, hypertension, and diabetes [21,22,23,24]. The Golgi apparatus processes, packages, and transports proteins and lipids. The lysosomes, ER, and Golgi apparatus have connected functions as part of a secretory pathway with the cell membrane; therefore, the subcellular targeting of sulfane sulfurs in these organelles using fluorescent probes is critical for better understanding the physiological and pathological impacts of sulfane sulfurs on various diseases. This greater knowledge may even have potential implications for clinical diagnosis and improved therapeutics.

**Figure 1:**
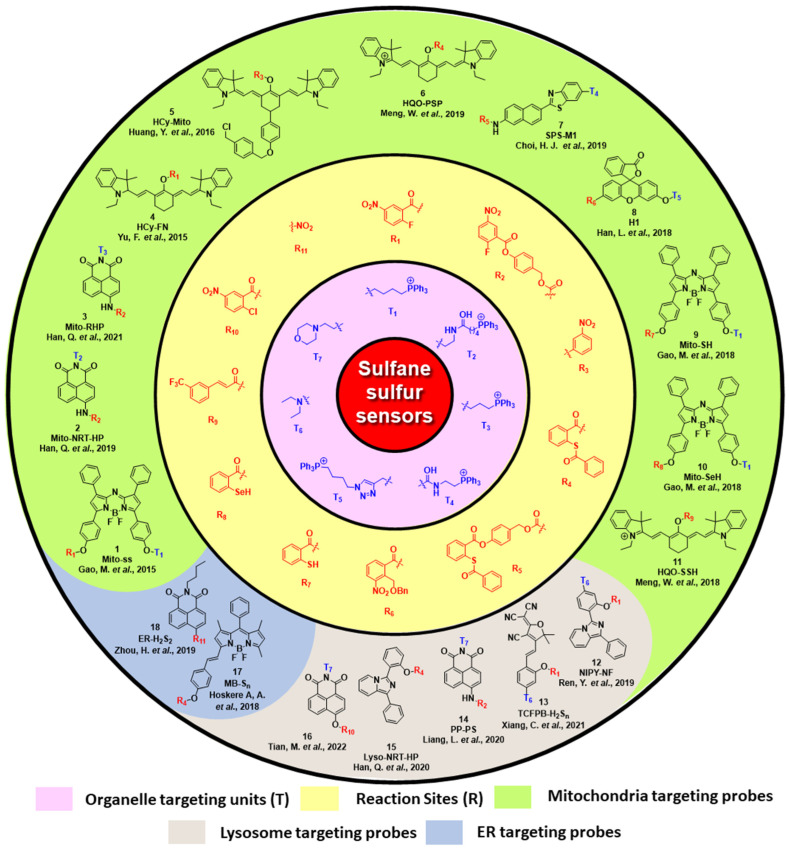
A summary of reported organelle-targeted fluorescent probes for sulfane sulfurs.

Due to the rising interest in organelles, sulfane sulfurs, and the role of sulfane sulfurs in maintaining intracellular redox homeostasis, developments in organelle-targeted fluorescent probes for sulfane sulfurs have been made in recent years. In this article, we reviewed the popular strategies for organelle-targeted probe design and discussed the reported organelle-targeted fluorescent probes for sulfane sulfurs along with their properties and potential limitations. A summary of these molecules is shown in Figure 1. 

## 2. Mitochondria-Targeting Probes 

Mitochondria are the major source of reactive oxygen species (ROS). During mitochondrial respiration, nearly 0.1–4% of oxygen is reduced to the superoxide ion (O_2_^•−^) due to electron leakage from the respiratory chain. This species is then transformed into other ROSs via enzymatic or non-enzymatic pathways [25]. Meanwhile, endogenously produced H_2_S is oxidized in the presence of mitochondrial ROS to form sulfane sulfurs, which can also be formed directly via enzymes such as 3MST. Thus, to better understand redox homeostasis, monitoring sulfane sulfurs via fluorescence imaging is useful. Mitochondria possess a unique double-layered membrane structure with a negative membrane potential (as high as −180 mV) [26]. Hence, in most cases, mitochondria-targeted probes possess at least one lipophilic cation [27]. Non-cationic probes can be functionalized by attaching triphenyl phosphonium [20] or pyridinium [28,29,30] as the anchor. However, functionalized cationic dyes are also known to target other organelles [31,32,33,34,35]. Based on colocalization experiments with commercially available mitochondria-targeting dyes, some non-cationic dyes have been reported to selectively target the mitochondria due to their unique structures [36]. Here, we summarize the reported cationic and non-cationic mitochondria-targeted probes for sulfane sulfur detection.

**Scheme 1:**
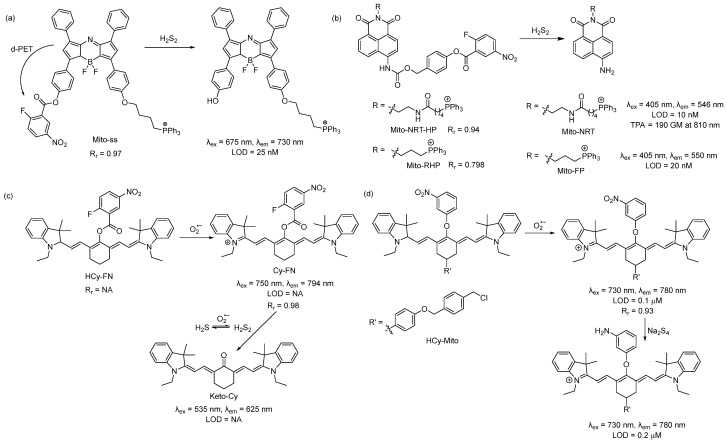
Structures and reactions of probes (**a**) Mito-ss, (**b**) Mito-NRT-HP, (**c**) HCy-FN, and (**d**) HCy-Mito.

In 2015, Chen and coworkers developed a reaction-based near-infrared (NIR) fluorescent probe (Mito-ss) for the detection of mitochondrial hydrogen polysulfides (H_2_S_n_, n > 1) [37]. Mito-ss consists of (i) a NIR dye based on the azo-BODIPY chromophore, (ii) a lipophilic triphenyl phosphonium group, and (iii) an H_2_S_n_-reactive nitrofluorobenzoate moiety (Scheme 1a). They chose a NIR fluorophore because NIR lights possess certain advantages, including deep tissue penetration, low cytotoxicity, and minimum background noise. Nitrofluorobenzoate is a commonly used functional group for the design of H_2_S_n_ sensors [38]. Nitrofluorobenzoate bears two electrophilic sites. H_2_S_n_ first reacts with it via nucleophilic aromatic substitution (S_N_Ar) to replace the F atom and form a persulfide (-SSH) intermediate, which then undergoes a spontaneous intramolecular cyclization with the ester group to uncage the fluorophore. Due to its electron with-drawing nature, nitrofluorobenzoate quenches the fluorescence of the azo-BODIPY chromophore via a donor-excited photoinduced electron transfer (d-PET) process. As such, Mito-ss is a reaction-based ‘turn-on’ sensor for H_2_S_n_. Mito-ss reacts rapidly (~30 s) with H_2_S_n_ and exhibits a 24-fold fluorescence increase at an emission of 730 nm. The probe was examined with various ROS, reactive nitrogen species (RNS), and other RSS and demonstrated no fluorescence turn-on. Biothiols such as glutathione (GSH), cysteine (Cys), *N*-acetyl-L-cysteine, etc., could react with Mito-ss. However, as the reaction stopped at the S_N_Ar step, no fluorescence was observed. The limit of detection (LOD) for Mito-ss was calculated to be 25 nM. The probe was used for the real-time detection of exogenous and endogenous H_2_S_n_ using six different cell lines. Mito-ss was also found to be suitable for the in vivo detection of exogenously injected H_2_S_n_ in BALB/c mice.

Using the same nitrofluorobenzoate reaction site, Han et al. developed a ratiometric fluorescent probe, Mito-NRT-HP, for the detection of mitochondrial H_2_S_n_ in 2019 [39]. The structure of Mito-NRT-HP is similar to Mito-ss, though with a two-photon responsive naphthalimide fluorophore instead of a single-photon responsive fluorophore. The naphthalimide fluorophore has the advantage of easily tunable photophysical properties by blocking and/or unblocking the internal charge transfer (ICT) process. It is highly photostable, resistant to pH interference, and possesses a large two-photon absorption cross-section. Importantly, 1,8-naphthalimide can be easily functionalized by simple synthetic tailoring [40]. The main advantages of two-photon excitation over single-photon excitation include deep tissue penetration, lesser damage, poor scattering, etc. In the case of two-photon excitation, a femto second pulsed laser is used, and the molecule can be excited only at the focal point of the laser. Three-dimensional imaging can be obtained [41]. Upon titrating with different concentrations of Na_2_S_2_, it was found that Mito-NRT-HP gave ratiometric responses with a changing fluorescence color from blue to green. When the solution of Mito-NRT-HP was treated with H_2_S_n_, the initial emission maximum at 478 nm decreased gradually, with a concomitant peak increase at 546 nm. The detection limit was 10 nM which suggests that Mito-NRT-HP could have the relevant sensitivity needed for the quantitative detection of H_2_S_n_ under physiological conditions. The two-photon absorption cross-section values (δ) of Mito-NRT-HP and its fluorophore Mito-NRT (Scheme 1b) were recorded in a buffer using a pulsed laser, and fluorescein was used as the reference molecule. Their δ values were measured over a range of wavelengths starting from 750 nm to 825 nm. The highest δ was 290 GM [1 GM (Goeppert-Mayer) = 10^−50^ cm^4^ s photon^−1^] for Mito-NRT-HP and ~190 GM for Mito-NRT at 810 nm. Mito-NRT-HP was found to exhibit good cell permeability and weak cytotoxicity, which was suitable for the ratiometric imaging of endogenous H_2_S_2_ in cells. Mito-NRT-HP was colocalized with MitoTracker Red (MTR) and LysoTracker Red (LTR), and the colocalization coefficients were found to be 0.94 and 0.42, respectively, indicating that Mito-NRT-HP was specifically localized in the mitochondria. Using two-photon microscopy, images of the tissue slices from mice with lipopolysaccharide (LPS)-induced acute organ injury were taken and compared with the control tissues. The enhanced fluorescence in the former case was observed. In 2021, Han et al. reported a similar probe for the detection of mitochondrial H_2_S_n_ during H_2_O_2_-induced redox imbalance [42]. The structure of this probe (Mito-RHP) only differed from Mito-NRT-HP in the linker between naphthalimide and the triphenylphosphonium unit. Upon the addition of Na_2_S_2_ to the solution of Mito-RHP, the initial emission spectra of Mito-RHP at 485 nm gradually decreased, and a continuous increase in the new peak to 550 nm was observed, along with a change in fluorescence color from blue to yellowish green. In this case, the Stokes shift was 109 nm, which was higher than that of Mito-NRT-HP. The detection limit was calculated to be 20 nM. Other properties, such as photostability, solubility, permeability, and cytotoxicity, were similar. However, the mitochondria-targeting ability of the new probe (overlap coefficient = 0.836) was not as good as that of Mito-NRT-HP (overlap coefficient = 0.94). The in vivo imaging of exogenous H_2_S_n_ (using Na_2_S_2_) was performed in zebrafish using Mito-RHP.

An interesting single-component multi analyte responsive NIR fluorescent probe was reported by Chen and coworkers in 2015 for the detection of the superoxide ion (O_2_^•−^) and H_2_S_n_ to understand redox homeostasis in the mitochondria [43]. Both O_2_^•−^ and H_2_S_n_ are short-lived reactive species, and their concentrations change quickly. To solve this problem, they developed a cyanine-based NIR probe, HCy-FN. This probe consists of two different reaction sites: one for the abstraction of hydrogen to detect O_2_^•−^ and the other for the detection of H_2_S_n_ using nitrofluorobenzoate (Scheme 1c). Both sensing steps were monitored by two different channels. Upon reacting with O_2_^•−^, HCy-FN was oxidized to Cy-FN, and this transformation was monitored by an increase in the emission intensity from channel 1 at 794 nm (λ_ex_ = 750 nm). Next, the nitrofluorobenzoate part of Cy-FN reacted with H_2_S_n_ to result in a decrease in the emission intensity of channel 1 followed by an increase in the emission intensity in channel 2 at 625 nm (λ_ex_ = 535 nm) due to the formation of Keto-Cy. They examined different ROS with HCy-FN and found that only O_2_^•−^ was able to oxidize the probe. Similarly, the reactivity of other RSS towards Cy-FN was also evaluated, and no changes in emission spectra were noted. HCy-FN was used for the detection of exogenous and endogenous H_2_S_n_ with the macrophage cell line RAW264.7 to monitor both sensing steps by dual channel emission. It was found that the Pearson correlation coefficient (R_r_) of Cy-FN and mitochondria-localizing Rhodamine 123 was 0.98, confirming that Cy-FN was localized in the mitochondria. Moreover, HCy-FN could detect endogenously produced O_2_^•−^/H_2_S_n_ in BALB/c mice. This work represents an interesting way to detect O_2_^•−^/H_2_S_n_ in the biological system. However, the claim that the probe is capable of monitoring mitochondrial O_2_^•−^/H_2_S_n_ may not be accurate. The authors only provided the R_r_ value for the intermediate compound Cy-FN and not for the actual probe. The structure of HCy-FN suggests that it may not be a suitable candidate to target the mitochondria because of the lack of a lipophilic cationic moiety.

In 2016, Chen and coworkers developed a probe (HCy-Mito) for the selective detection of superoxide anion (O_2_^•−^) and H_2_S_n_ in the mitochondria [44]. The reaction sites for O_2_^•−^ and H_2_S_n_ were the reduced cyanine dye (similar to HCy-FN) and *m*-nitrophenyl ether (Scheme 1d). In the presence of O_2_^•−^, Hcy-Mito was oxidized to form a cyanine derivative, and the reduced nature of H_2_S_n_ converted the nitro group to -NH_2_, which terminated the d-PET process and resulted in an increase in emission intensity to 780 nm. The detection limits for O_2_^•−^ and H_2_S_n_ by HCy-Mito were found to be 0.1 μM and 0.2 μM, respectively. In vitro experiments with RAW264.7 cells by HCy-Mito suggest that it could image exogenous and endogenous O_2_^•−^/H_2_S_n_ and localize specifically in the mitochondria (R_r_ = 0.93). This probe was further utilized for the in vivo detection of O_2_^•−^ (generated from phorbol myristate acetate (PMA) and H_2_S_n_ (via injected Na_2_S_4_) in BALB/c mice.

**Scheme 2:**
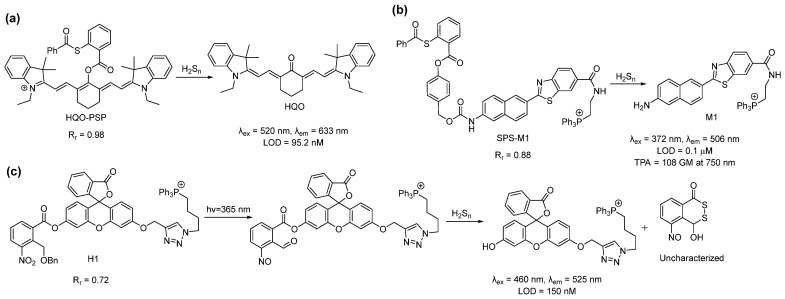
Structures and reactions of probes (**a**) HQO-PSP, (**b**) SPS-M1, and (**c**) H1.

In 2019, Meng et al., used a different reaction site based on the 2-(acylthio)benzoate for the design of a mitochondria-targeted probe for H_2_S_n_ [45]. This template utilized both the nucleophilic and electrophilic nature of H_2_S_n_ for its recognition (Scheme 2a) [46]. Briefly, the thioester exchange between the H_2_S_n_ and 2-(acylthio)benzoate produced a thiophenol derivative, which, in turn, reacted with H_2_S_n_ to form an -SSH intermediate. This intermediate underwent an intramolecular cyclization to release the fluorophore. This template was attached to a red-emitting fluorophore to develop the probe, HQO-PSP. HQO-PSP itself was non-fluorescent but, upon sensing H_2_S_n_, exhibited a fluorescence turn-on (86-fold) at an emission of 633 nm due to the formation of the keto derivative HQO. The probe was found to be relatively fast (7 min) and highly selective to H_2_S_n_, with a detection limit of 95.2 nM. In vitro studies with A549 cells revealed that HQO-PSP could specifically localize within the mitochondria (R_r_ = 0.98) and selectively image exogenously added H_2_S_n_ in the live cells.

In the same year, Choi et al. reported that the ratiometric probe SPS-M1 for mitochondrial H_2_S_n_ detection was based on a two-photon excitable naphthalene fluorophore [47]. The reaction site was the same as that of HQO-PSP except for an additional self-immolating carbamate linker (Scheme 2b). The probe exhibited a blue fluorescence (λ_em_ = 429 nm) but produced the deprotected yellow fluorescent dye M1 (λ_em_ = 506 nm) upon sensing H_2_S_n_. Interestingly, the two-photon absorption (TPA) cross-section (δ) of SPS-M1 and M1 was found to be 11 and 108 GM, respectively, at 750 nm. The large TPA cross-section resulted from the strong ICT process in M1. SPS-M1 was found to be suitable for the quantification of H_2_S_n_ in live cells, and the in vitro detection limit was 1 μM. The two-photon microscopic imaging with SPS-M1 for endogenous H_2_S_n_ using the wild-type and Parkinson’s disease (PD) model neurons and brain tissues of mice revealed that H_2_S_n_ concentrations were higher in the PD model. 

Another interesting approach for the spatiotemporal detection of mitochondrial H_2_S_n_ was reported by Han et al. in 2018 [48]. Probe H1 consisted of a fluorescein dye attached to a triphenylphosphonium group and a nitrobenzyl photoactivable protecting group (Scheme 2c). Upon irradiation with UV light (365 nm), the nitrobenzyl part produced an aldehyde derivative, which served as the H_2_S_n_ recognition site. H_2_S_n_ attacks the aldehyde group to form a persulfide intermediate, which then should undergo cyclization to liberate the fluorophore and generate a side product (4-hydroxybenzo[*d*][1,2]dithiin-1(4*H*)-one). H1 showed a turn-on of fluorescence at 525 nm only when it was irradiated with UV light along with H_2_S_2_ in the solutions. The detection limit was calculated to be 150 nM. The targeting ability of H1 was confirmed by counterstaining with MitoTracker Green (MTG) (R_r_ = 0.72). Although this photo-triggered probe was interesting, the authors did not provide experimental support for the proposed detection mechanism. This aldehyde-based intermediate may also possess some problems as 2-formyl carboxylate is a well-known H_2_S recognition site [49,50], and the aldehyde group has a high reactivity towards free cysteine [51,52,53].

**Scheme 3:**
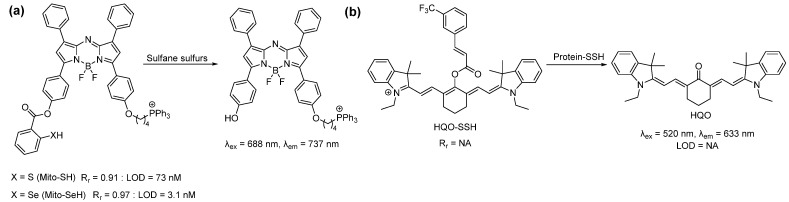
Structures and reactions of probes (**a**) Mito-SH/Mito-SeH and (**b**) HQO-SSH.

In addition to specific probes for H_2_S_n_, general probes for sulfane sulfurs in the mitochondria have also been reported. Chen and coworkers reported the sulfane sulfur-responsive probe Mito-SH based on the azo-BODIPY fluorophore with a sulfane sulfur reaction site-thiosalicylate (Scheme 3a) in 2018 [54]. The sensing mechanism was based on the nucleophilicity of the thiol group toward the electrophilic sulfane sulfurs [55,56]. Upon reacting with sulfane sulfurs, the probe formed an -SSH intermediate, which immediately underwent an intermolecular cyclization to release the fluorophore. Mito-SH was found to be highly selective, highly responsive (100 s), and free from pH interference (range pH 4–7.8). It exhibited a 10-fold enhancement in the emission intensity upon sensing sulfane sulfurs in the NIR region (723 nm). The detection limit was 73 nM. Mito-SH exhibited mitochondria-specific localization as verified by a colocalization experiment with MTG (R_r_ = 0.91). In vitro imaging of exogenous (with Na_2_S_4_ as the source) and endogenous (generated from CSE) sulfane sulfurs were performed with this probe using SH-SY5Y cells. This was then further utilized for imaging sulfane sulfur changes caused by acute ischemia in mice. The same group reported a different approach in 2018, utilizing the reactivity of the selenol (-SeH) group towards sulfane sulfurs [57]. The probe Mito-SeH is the same as Mito-SH except for the replacement of -SH by -SeH. Due to the difference in the p*K*_a_ value of SeH (p*K*_a_ 5.9) vs. SH (p*K*_a_ 6.5), the former was found to be more reactive towards sulfane sulfurs. Mito-SeH ratiometrically reacted with sulfane sulfurs and exhibited fluorescence when turned on at 720 nm with a detection limit of 3.1 nM. In vitro experimentation using smooth muscle cells (SMCs) revealed that this probe could sense both exogenous (using Na_2_S_4_, thiophosphates, or 3H-1, 2-dithiole-3-thione as the source of sulfane sulfurs) and endogenous sulfane sulfurs (using LPS to induce cystathionine γ-lyase (CSE) production). Mito-SeH was utilized for the in vivo detection of sulfane sulfurs in the acute ischemia of mice, and it was concluded that sulfane sulfurs exhibited cytoprotective effects against hypoxia. While this selenol-based probe showed interesting activities, its stability could be a problem as -SeH groups are known to be highly sensitive to oxidation under air. 

In 2018, Meng et al. reported an ‘off-on’ fluorescent probe HQO-SSH for the detection of protein persulfidation. This probe appeared to also target mitochondria (presumably due to the cationic nature of the cyanine dye) [58]. The authors claimed that this probe was selected by screening a library of compounds to identify a suitable functional group to specifically react with persulfides. However, it is unclear what compounds were screened. It was suggested that persulfides could remove the acryloyl group and release the cyanine dye while other species, such as H_2_S, biothiols, ROS, etc., could not. Persulfidated papain and glyceraldehyde-3-phosphate dehydrogenase (GAPDH) were used as models to validate the probe. Upon sensing persulfides, it exhibited a turn-on of fluorescence at ~635 nm. The probe was used for the imaging of mitochondrial protein persulfide changes in A549 and BEAS-2B lung cells (persulfidation induced by propargylglycine and Na_2_S), as well as in sulfur mustard-induced lung injury tissues. However, those studies did not rule out the possibility of the probe turn-on by non-protein persulfides, such as small molecule persulfides or other sulfane sulfur species. 

## 3. Lysosome-Targeted Probes

Lysosomes are membrane-bound organelles with acidic pH values which could reach as low as 4.5–4.7 [59]. A popular method to target these vesicles involves exploiting their low pH by incorporating a moiety (normally lipophilic amines) that can become easily protonated on a fluorescent sensor upon entering the lysosome. [60] The resulting compound is membrane impermeable and can then accumulate in the acidic lysosomal matrix. However, it must be noted that not all lysosome-targeted fluorescent probes contain a pH-sensing moiety [61,62,63].

**Scheme 4:**
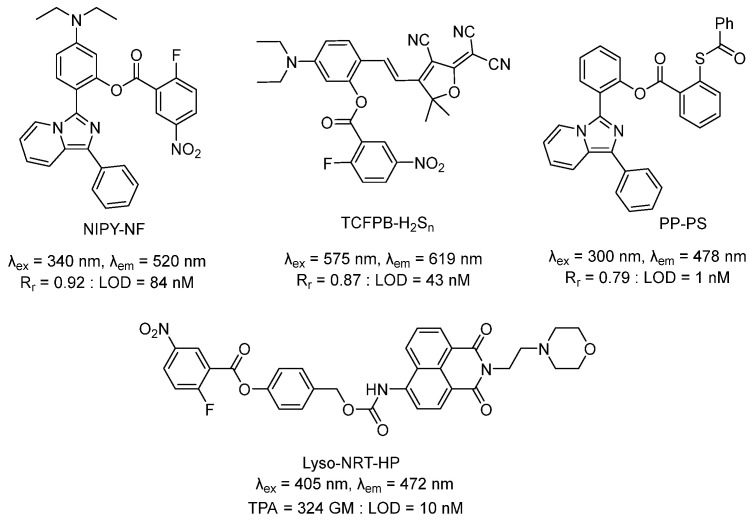
Structures of lysosome-targeted probes.

One of the first lysosome-targeted fluorescent probes for sulfane sulfur detection was reported by Ren et al. in 2019 [64]. They utilized diethylamine to direct the probe to the lysosomes. The previously reported imidazo [1,5-α] pyridine derivative NIPY-OH fluorophore was chosen for its large Stokes shift (215 nm) due to its excited-state intramolecular proton transfer (ESIPT) process [65]. This property decreases the issues of self-quenching and auto-fluorescence, which can impact probe performance. The popular 2-fluoro-5-nitrobenzoic ester moiety was selected as the analyte recognition site. The combination of these three groups resulted in the probe NIPY-NF (Scheme 4). Due to photoinduced electron transfer (PET), the probe itself was non-fluorescent. UV-vis and fluorescence analyses determined that NIPY-NF, after responding to H_2_S_2_, had an excitation wavelength of 340 nm, an emission of 520 nm, a detection limit of 84 nM, and a quick response time (<6 min). It was also observed that NIPY-NF was applied to A549 cells with low cytotoxicity. Bioimaging studies in this cell line determined that the probe could exogenously detect H_2_S_n_ in the cells after treatment with Na_2_S_2_ as well as after LPS stimulation to increase endogenous levels of H_2_S_n_. The probe’s ability to localize in the lysosomes was confirmed through a colocalization study with the probe and LysoTracker Green.

The same lysosome targeting and H_2_S_n_ detection strategies were employed by Xiang et al. in 2021 to develop a ratiometric fluorescent probe, TCFPB-H_2_S_n_, with aggregation-induced emission (AIE) characteristics for in vitro and in vivo applications [66]. The benefits of sensors with AIE include the ability to overcome aggregation-induced quenching (ACQ) issues (i.e., decreased fluorescence, autofluorescence in vivo, etc.) that are common to ratiometric probes. This probe employed a tricyanofuranyl imino-salicylaldehyde (TCFIS) as the fluorophore with the incorporation of the 2-fluoro-5-nitrobenzoate to allow for an ICT. Weak ICT effects were expected due to the ester group’s weaker electron-donating ability compared to that of the phenol. While the probe itself was expected to be somewhat fluorescent due to π-conjugation in the chain despite the addition of the 2-fluoro-5-nitrobenzoate, the presence of H_2_S_n_ was expected to yield strong ICT effects and lead to an enhanced fluorescence based on DFT calculations. The AIE characteristic, mechanism of the probe, and ICT occurrence (ex: 575 nm; em: 619 nm increase after Na_2_S_4_ addition; em: 751 nm decrease after Na_2_S_4_ addition) were verified. Further analyses determined the limit of detection (43 nM) along with a fast reaction time (2 min) and the specificity of the probe for H_2_S_n_. The intensity of the ratio for the fluorescence signal (*I*_619_*/I*_751_) decreased at lower pHs (3–5) relative to those at pHs 5–10. Considering the weakly acidic environment of the lysosome, this had the potential to affect probe efficacy. The authors also determined TCFPB-H_2_S_n_’s applicability in biological systems. It was found that the probe had low cytotoxicity (5–25 μM) in HeLa cells, and TCFIS could localize in the lysosomes though with some fluorescence in other areas of the cell (R_r_ ~0.87217). Significantly, the probe was capable of the real-time imaging of H_2_S_n_ in mice models of acute ulcerative colitis.

Though the 2-fluoro-5-nitrobenzoate-based probes demonstrated good selectivity, the high reactivity of the fluorobenzene had the potential to cause the probes to be consumed in the presence of biothiols. In 2020, Liang et al. reported PP-PS (2-(1-phenylimidazo[1,5-α]pyridin-3-yl)phenyl-2-(benzoylthio)benzoate): [67] a turn-on probe using a previously reported fluorophore PP-OH [68] and a known 2-(acylthio)benzoate reaction site for H_2_S_n_ [46]. The fluorophore could likely be protonated due to its increased stability from aromaticity, thus promoting its aggregation in acidic environments such as the lysosome. The quantum yield of PP-PS was found to be 0.0132 (weak fluorescence signal), which increased to 0.12549 (em: 478 nm, ex: 300 nm) upon the addition of Na_2_S_2_. Fluorescence analyses determined the limit of detection (1 nM) and a 1 min response time. The probe was found to have low cytotoxicity in A549, MCF-7, and U87 cancer cells, and the fluorescence cell imaging of H_2_S_n_ was successful in these cell lines. The probe was also applied in xenograft mouse tumor tissues and LPS-induced inflammation in excised mouse tissues. Colocalization studies using LPS-treated A549 cells, PP-PS, and LysoRed confirmed the presence of the dye in the lysosomes (R_r_ = 0.79192).

In 2020, Han et al. utilized 4-(2-aminoethyl)-morpholine to prepare Lyso-NRT-HP: a lysosomal-targeted ratiometric two-photon fluorescent probe for H_2_S_n_ [69]. Their design also included a 1,8-naphthalimide based fluorophore and a 2-fluoro-5-nitrobenzoyl group to serve as the H_2_S_n_ receptor. The former is known for its effective ICT fluorescence and photophysical properties, such as photostability and large Stokes shift, and has been successfully used in the design of other ratiometric two-photon H_2_S_n_ probes [70]. The latter allows the probe to specifically react with H_2_S_n_ to release the fluorophore Lyso-NRT with a fluorescence emission of 548 nm in contrast to the 472 nm emission of Lyso-NRT-HP. Similarly, the absorption band of the probe was 384 nm, while Lyso-NRT’s was 432 nm. The two-photon (TP) induced fluorescence property of Lyso-NRT-HP was confirmed by determining the TPA cross sections of the probe itself and its product after it reacted with Na_2_S_2_ (δ = 324 GM). Analyses of the probe determined its selectivity towards H_2_S_n_ over other biologically relevant RSS, including stability, quick turn-on (5 min), and limit of detection (10 nM). Lyso-NRT-HP was then applied in HeLa cells and demonstrated its ability to image H_2_S_n_. A co-localization study was also carried out to determine whether the probe could be lysosome targeting. HeLa cells were treated with either the probe, LysoTracker Red DND-99, or both, and it was determined that Lyso-NRT could be observed in the lysosomes. Imaging studies were also performed on fresh kidney slices. A similar design was used by Tian et al. in 2022 with a 4-hydroxy-1,8-naphthalimide fluorophore, 2-chloro-5-nitrobenzoate group as the H_2_S_n_ recognition site, and the same morpholine moiety [71]. This probe was applied in HeLa cells for H_2_S_n_ detection.

## 4. ER-Targeted Probes

**Scheme 5:**
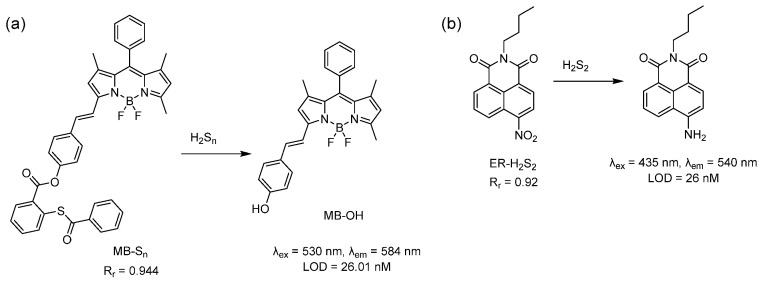
Structures and reactions of probes (**a**) MB-S_n_ and (**b**) ER-H_2_S_2_.

The redox state of the ER was dominated by RSS as the main location for protein disulfide bond formation [72]. Hence, targeting the ER for sulfane sulfur is important to understand the sulfane sulfur dynamics of living systems. Normally, the phenyl sulfonamide moiety is used as the ER-targeting unit because it can bind to cyclooxagenase (COX), which is abundant in the ER membrane [60]. To date, only two probes have been reported for containing imaging sulfane sulfurs within the ER [72,73]. However, neither of the probes is linked to any specific ER-targeting units. Based on colocalization experiments, they were found to be selectively localized within the ER. The first probe, MB-S_n_, was reported in 2018 by Das et al. and was found to detect H_2_S_n_ in the ER (Scheme 5a) [73]. This probe consisted of the well-known thiosalicylate recognition site for H_2_S_n_ and a BODIPY-based fluorophore. Upon sensing H_2_S_n_, MB-S_n_ exhibited a turn-on at an emission of 584 nm due to the formation of MB-OH with a detection limit of 26 nM. In vitro studies with MB-S_n_ using RAW264.7 cells confirmed its ability to sense LPS-induced endogenous sulfane sulfurs and exogenous Na_2_S_2_. The colocalization study of MB-S_n_ with ER-Tracker Green confirmed its ability to target the ER (R_r_ = 0.944).

Zhou et al. utilized the stronger reducing power of H_2_S_2_ compared to H_2_S towards the nitro group and developed a naphthalimide-based probe, ER-H_2_S_2_, in 2019 (Scheme 5b) [72]. Due to the presence of -NO_2_ in the probe, the fluorescence of the probe was masked. ER-H_2_S_2_ exhibited an enhanced fluorescence at 540 nm with a large Stokes shift of 105 nm in the presence of Na_2_S_2_. The observed fluorescence could be attributed to the reduction from -NO_2_ to -NH_2,_ which then participated in the ICT process. The detection limit was 26 nM. The selectivity of ER-H_2_S_2_ was checked against different ROS, RNS, and RSS, including H_2_S_,_ and all yielded little to no fluorescence. It should be noted that very similar nitro-containing naphthalimide-based probes have been reported to sense H_2_S [74,75]. Thus, these results may appear controversial. It was reported that lipophilic compounds such as alkyl chain-appended naphthalimides tended to accumulate in the lipid-dense ER region. The ER-targeting ability of ER-H_2_S_2_ was confirmed by a tracking experiment with ER-Tracker Red (R_r_ = 0.92). This probe was used to detect exogenous H_2_S_2_ in zebrafish. 

## 5. H_2_S_2_-Triggered Drug Delivery

**Scheme 6:**
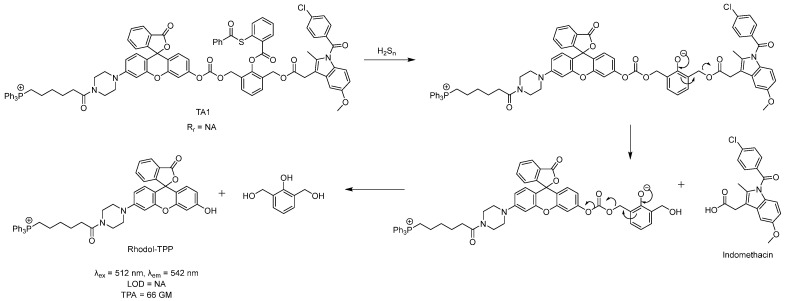
The structure and mechanism of TA1.

Considering the increasingly recognized role of sulfane sulfurs in maintaining cellular redox homeostasis and the recently developed approaches that can specifically sense these analytes in biological systems, it may be possible to utilize elevated sulfane sulfur levels as markers for disease diagnostics and targeted drug delivery. For example, Kim et al. developed a theranostic agent (TA1) in 2021 that could selectively sense H_2_S_n_ while simultaneously releasing an anti-inflammatory drug (Scheme 6) because H_2_S_n_ is an inflammatory site biomarker that can be stored in the mitochondria [76]. TA1 consists of an H_2_S_n_ recognition site (2-(acylthio)benzoate), a two-photon responsive rhodol fluorophore-appended triphenylphosphonium unit (Rhodol-TPP), the anti-inflammatory cyclooxygenase enzyme (COX) inhibitor (indomethacin), and a self-immolating linker. Upon reacting with H_2_S_n_, TA1 released both the fluorophore and drug. This step was monitored by the increase in TA1’s fluorescence intensity at 542 nm, which provided real-time information about the release of indomethacin. In H_2_S_n_ overexpressed models, TA1 suppressed both the COX-2 level in live cells and the prostaglandin E_2_ (PGE_2_) level in blood serum. Thus, TA1 may be considered an inflammation site-selective theranostic agent for precise diagnosis and anti-inflammatory therapy.

## 6. Conclusions

The development of fluorescent spectroscopy and fluorescent microscopy technologies over the years has enabled advancement in the understanding of various biochemical processes that occur in biological systems. For example, cellular sulfane sulfurs have received increasing recognition as an important class of RSS that play key roles in multiple physiological and pathological processes [2,3,4,5]. Yet, despite the generally acknowledged functional importance of subcellular organelles to the health of the overall cell, organelle-targeted fluorescent probes for sulfane sulfur detection are underexplored. Major challenges include the inherent instabilities of RSSH and H_2_S_n_, the difficulties of specifically targeting individual organelles, and the sensitivities required to detect sulfane sulfurs at the subcellular level with the expectation that various organelles have different levels of endogenous sulfane sulfurs. In this review, we summarized the sulfane sulfur sensing mechanisms of reported organelle-targeted sensors, their targeting abilities as demonstrated through colocalization studies (based on the calculated Pearson correlation coefficient), sensitivities (by the limit of detection), applicability towards sensing endogenous and exogenous sulfane sulfurs under physiological conditions, and their advantages/disadvantages for the chosen fluorophores. While most of the reported (yet admittedly limited) organelle-targeted sulfane sulfur sensors were designed to target the mitochondria, only a few were synthesized to target the lysosomes and ER. Most of the reported sensors were rationally designed, but some sensors lacked organelle-targeting anchors. As such, the accumulation of these sensors and fluorophores in their desired organelles may possibly be attributed to their unique structural characteristics rather than their targeting ability. Increased development of more diverse and selective organelle-targeting groups that can couple to fluorescent sensors would greatly enhance knowledge in the field. Additionally, to the best of our knowledge, organelle-targeted sulfane sulfur probes for other organelles (i.e., Golgi apparatus, nucleus, etc.) have yet to be developed and provide a potential direction for future work.

Other concerns with some of the probes mentioned in this review involve their sulfane sulfur reaction sites because they have been used for the detection of other analytes, such as H_2_S. This raises issues regarding the specificity of the sensors for sulfane sulfurs. The sensing mechanisms of some probes were also not reported. As such, areas of exploration for future work include the discovery of novel and more specific reactions for sulfane sulfur. Through the development and improvement of chemical tools to detect sulfane sulfurs in subcellular organelles, we expect to gain an increased understanding of the role sulfane sulfurs play in the biological system. This may, in turn, lead to the advancement of highly valuable sulfane sulfur-based theranostics. In summary, we expect to see more interesting works from this field in the near future.
